# Society Meetings

**Published:** 1905-07

**Authors:** 


### SOCIETY MEETINGS.

THE International Society of Surgery will hold its first meeting at Brussels, September, 1905, under the presidency of Professor Th. Kocher, of Berne. All communications relating to the congress should be addresses to Dr. Ch. Willems, 6 Place, Michel, Brussells.

---

THE American Electrotherapeutic Association will hold its fifteenth annual meeting at the Academy of Medicine, New York, September 19, 20, and 21, 1905. Address all communications to Dr. Clarence E. Skinner, secretary, 67 Grove street, New Haven, Conn.

---

THE Physicians' League of Buffalo held its annual meeeting Wednesday evening, May 31, at the Riverside hospital, and elected the following officers: president, Dr. Jane W. Carroll; vice-president, Dr. Janet Himmelsbach; secretary and treasurer, Dr. Katherine Munhall.

The league held its annual banquet at the Lafayette hotel Tuesday evening, June 13, 1905. Dr. Jane W. Carroll, president of the league, acted as toastmaster. Dr. Janet Himmelsbach responded to the toast, "The Medical Woman as a Physician," and Dr. Marie Wolcott spoke of "The Medical Woman as a Teacher." Dr. Mary I. Denton's topic was "As President to President," and Dr. M. Elizabeth Schugens responded to the toast, "The Medical Woman as a Woman."

---

THE Buffalo Academy of Medicine held meeting during the month of June, 1905, as follows:

> The annual meeting of the academy was held at the academy rooms, Public Library building, Tuesday evening, June 13, 1905. Program: President's address: The uses of a medical society, Dr. Arthur W. Hurd. Election of officers was held for the ensuing year.

